# Correlation Between TIMI Risk Score and the Number of Vessels Involved in the Angiographic Study; a Cross-sectional Study 

**DOI:** 10.22037/aaem.v10i1.1466

**Published:** 2022-02-14

**Authors:** Mohammad Hasan Namazi, Seyede Salimeh Mazloomi, Mohammad Kalate Aghamohammadi

**Affiliations:** 1 Department of Cardiology, Moddares Hospital, Shahid Beheshti University of Medical Sciences, Tehran, Iran.; 2 Department of Internal Medicine, Shohadaye Tajrish Hospital, Shahid Beheshti University of Medical Sciences, Tehran, Iran.

**Keywords:** Myocardial Infarction, Risk Assessment, Coronary Circulation, Non-ST Elevated Myocardial Infarction

## Abstract

**Introduction:**

TIMI (Thrombolysis in Myocardial Infarction) score is a model for predicting the severity of vascular diseases. This study aimed to evaluate the correlation between this score and the number of vessels involved in patients with Unstable Angina (UA) or Non-ST Elevation Myocardial Infarction (NSTEMI).

**Methods:**

This prospective cross-sectional study was designed to evaluate the correlation between TIMI score, and the number of vessels involved in the angiographic study of NSTEMI and UA patients presenting to emergency department.

**Results:**

297 patients with the mean age of 62.16±36.59 years were entered (58.2% male; 193 (65%) UA and 104 (35%) NSTEMI). The Mean TIMI score among patients was 3.21±1.55. Based on the TIMI score, patients were categorized into 3 groups. 105 (35.35%) patients had a TIMI score of 0 to 2, 120 (40.40%) had a score of 3 to 4, and 72 (24.24%) had a score of 5 to 7. Patients with a TIMI score of 5 to 7 had a greater likelihood of three-vessel coronary artery disease compared to patients with a TIMI score of 3 to 4 (OR: 5.34, 95% CI: 2.64 to 10.80; p < 0.0001) or those with a TIMI score of 0 to 2. (OR: 29.45, 95% CI: 12.87 to 67.37; p < 0.0001). Two-vessel coronary artery disease was more likely to be found in patients with a TIMI score of 3 to 4 or those with a score of 5 to 7 compared to patients with a TIMI score of 0 to 2 (OR: 3.69, 95% CI: 1.60 to 8.51; p <0.0001 and OR: 2.67, 95% CI: 1.04 to 6.82; p = 0.04, respectively).

**Conclusion:**

There is a direct and significant correlation between TIMI score and the number of coronary vessels involved in patients presenting to emergency department following UA or NSTEMI.

## 1. Introduction:

Advancements of cardiac care units and revascularization methods, as well as the developments in pharmacotherapy, have led to improved patient outcomes after acute coronary syndrome (ACS) (1, 2). Despite recent developments, coronary artery disease (CAD) remains the leading cause of death across the world. Myocardial infarction (MI) is known as the most severe presentation of CAD and CAD accounts for 30% of all mortalities (3). Each year, three million people experience ST-segment elevation MI (STEMI) and also non-ST-segment elevation MI (NSTEMI) was estimated to occur in about four million (4). Several randomized clinical trials performed in the past two decades have established that immediate and complete restoration of flow in the occluded artery decreases infarct size, improves survival rates, and preserves left ventricular (LV) function (5, 6). Choosing the best treatment in the initial stages following quick diagnosis is of great importance in improving the outcome. Nonetheless, electrocardiogram (ECG), as the most readily available diagnostic tool in chest pain units, is not adequately helpful in decision making(7). The TIMI (Thrombolysis in Myocardial Infarction) research group has introduced a specific model, the TIMI risk score assessment tool, which has been found to be predictive of the severity of vascular diseases and the potential of coronary circulation involvement in chest pain patients (8). There are seven components used in the calculation of the TIMI score. Patients presenting with Unstable Angina (UA) or NSTEMI that fit score 3 or more on the TIMI model are mostly recommended to undergo early invasive management with cardiac angiography and revascularization if necessary (9). 

The TIMI score was established as one of the most commonly utilized risk assessment models in the chest pain units to warrant further workup. Hence, the development of the TIMI risk score originated from developed countries, with limited data evaluating the effectiveness in developing countries. This is particularly important for lower-middle-income countries, which are increasingly affected by cardiovascular disease epidemic and encompass different genetics and lifestyle. This study aimed to evaluate the correlation between TIMI risk score and the number of vessels involved in the angiographic study of patients presenting to emergency department following UA or NSTEMI.

## 2. Methods:

***2.1 Study design and setting***

This prospective cross-sectional study was designed to evaluate the correlation between TIMI score, and the number of vessels involved in the angiographic study of NSTEMI and UA patients, presenting to Modarres Hospital, Tehran, Iran, from April 2019 to August 2020. The study was approved by the ethics committee of Shahid Beheshti University of Medical Sciences (Code: IR.SBMU.RETECH.REC.1398) and the researchers adhered to the principles of the Helsinki Declaration and patient data confidentiality throughout the study. Written informed consent was obtained from all the patients for participating in the study. 

***2.2. Participants***

Patients with UA or NSTEMI who underwent angiographic study were included in the study using census sampling method. Patients with unclear past medical history or past coronary angiographic history, prior coronary artery bypass graft (CABG), atypical chest pain, evidence of ST-segment elevation in the initial ECG, those who underwent the angiographic procedure due to reasons other than ischemic heart disease, and those who did not give consent for participating were excluded.

***2.3. Data gathering***

Data on baseline characteristics, including demographics (age, sex), risk factors (weight, hypertension, diabetes, current smoking, family history of coronary artery disease), and medical history (prior angina or MI), were collected. TIMI score predictive factors (Presence of at least three risk factors for coronary artery disease, age of 65 years or older, presence of 2 or more episodes of angina 24 hours before the presentation, aspirin use in the past seven days, previous history of coronary stenosis of 50% or more, ST-segment deviations greater than or equal to 0.05 mV on initial ECG on admission, elevated serum cardiac markers of necrosis) and angiographic data were also included in the checklist.

Coronary artery angiography was done and reported by one expert cardiologist. Stenosis of the coronary artery was given a score of 0 if narrowing was less than 50% and a score of 1 if stenosis was greater than 50%. Echocardiography study was done by an expert cardiologist at the time of admission using vivid^® ^S6 GE device. Cardiologists who performed the angiographies and echocardiographs were blinded to the TIMI scores of patients.

**2.4. TIMI score**

The following seven factors help assess the mortality risk:

1. Presence of at least three risk factors for coronary artery disease (i.e., diabetes mellitus, hypertension, hyperlipidemia, smoking, family history).

2. Age of 65 years or older.

3. Presence of 2 or more episodes of angina 24 hours before the presentation.

4. Aspirin use in the past seven days.

5. Previous history of coronary stenosis of 50% or more.

6. ST-segment deviations more than or equal to 0.05 mV on ECG on admission.

7. Elevated serum cardiac markers of necrosis.

Each factor has a value of one point. A higher score implies a higher likelihood of mortality.

The risk of mortality and need for further invasive cardiac intervention for each score is as follows:

- 4.7% for a score of 0/1
- 8.3% for a score of 2
- 13.2% for a score of 3
- 19.9% for a score of 4
- 26.2% for a score of 5
- 40.9% for a score of 6/7

Scores ranging from 0-2 represent a low risk. Scores from 3-5 are considered to point to intermediate risk. A score of 6 or 7 indicates high risk (10).

***2.5. Statistical analysis***

Patient characteristics were summarized using frequency (%) or mean ± standard deviation. Logistic regression analysis was used to assess the correlation between the TIMI risk and severity of CAD. Results are presented as odds ratios (ORs) with corresponding 95% confidence intervals (CIs) and IBM SPSS 23.0 was used to perform statistical analyses. (IBM SPSS Inc., Chicago, IL). For all analyses, two-sided P values <0.05 were considered significant.

## 3. Results:

***3.1. Baseline characteristics of studied patients***

297 patients with mean age of 62.16±36.59 years who presented with acute coronary syndrome and underwent coronary angiography during the study period were entered (58.2% male). 193 (65%) were diagnosed with UA and 104 (35%) with NSTEMI. The Mean ejection fraction within the study population was 48.68%±10.25%. Among the studied cases, 27 (9%) patients were categorized as having severe systolic dysfunction with an EF of 30% or less. The Mean number of significant coronary lesions was 1.75±1.18 vessels. 26 (8.8%) patients had ectatic coronary vessels in angiography. Tables 1 and 2 show the baseline characteristics of the patients. The Mean TIMI score among patients was 3.21±1.55. Based on the TIMI score, patients were categorized into 3 groups. 105 (35.35%) patients had a TIMI score of 0 to 2, 120 (40.40%) had a score of 3 to 4, and 72 (24.24%) had a score of 5 to 7. 

***Correlation of TIMI score and number of vessels involved***

Table 3 summarizes the correlation between TIMI score and the number of coronary arteries involved. Patients with a TIMI score of 5 to 7 had a greater likelihood of three-vessel coronary artery disease compared to patients with a TIMI score of 3 to 4 (OR: 5.34, 95% CI: 2.64 to 10.80; p < 0.0001) or those with a TIMI score of 0 to 2 (OR: 29.45, 95% CI: 12.87 to 67.37; p < 0.0001). Two-vessel coronary artery disease was more likely to be found in patients with a TIMI score of 3 to 4 or those with a score of 5 to 7 compared to patients with a TIMI score of 0 to 2 (OR: 3.69, 95% CI: 1.60 to 8.51; p <0.0001 and OR: 2.67, 95% CI: 1.04 to 6.82; p = 0.04, respectively).

One-vessel CAD was also seen more often in patients with a score of 0 to 2 compared to patients with a score of 3 to 4 or 5 to 7 (OR: 15.34, 95% CI: 3.54 to 66.42; p< 0.0001 and OR: 13.83, 95% CI: 3.21 to 59.61; p < 0.0001). No statistically significant correlation was found between non-significant CAD angiographic finding and TIMI score.

## 4. Discussion:

Patients with NSTEMI are older and have multiple cardiovascular risk factors, as well as an increased risk of cardiovascular complications. Hence, the TIMI risk score was found to be helpful in the identification of high-risk patients. This study was conducted to assess the correlation of the number of occluded vessels with TIMI risk score. The main findings of this study demonstrated that TIMI score appears to be a practical tool for identifying high-risk patients after AMI, particularly in relation to the number of vessels with thrombus, and can also predict the findings in angiography follow-up. 

In this study, data were collected from a group of 297 patients who presented to our center with signs or symptoms of UA or NSTEMI; their TIMI score calculated at the time of admission significantly correlated with the number of occluded vessels and severity of ischemia later found in the angiographic study (Average TIMI score was 3.2 vs. average number of occluded vessels 1.8). Most patients were men younger than 65 years old and diagnosed with unstable angina. Our study revealed that TIMI score significantly increased with an increase in the number of vessels involved in thrombotic obstruction (P-value=0.001, r= 0.639). The correlation was found in both groups of UA and NSTEMI.

TIMI score has been validated by the TACTICS-TIMI trial (11) and PRISM-PLUS trial (12). Also, based on the ESSENCE trials (13) and TIMI IIB (9) study, the TIMI risk score incorporates the predictive factors of clinical characteristics, ECG changes, and cardiac biomarkers for risk assessment. This score also identifies high-risk patients and determines whether an immediate invasive strategy would be beneficial in such patients (14). Garcia et al. demonstrated that the score has broader usefulness in risk stratification and is easy to use as a bedside tool (15). In their experience, a cutoff of 5 and more in TIMI score distinguished patients with 3-vessel or left main disease from those without these diseases. However, the final decision regarding which revascularization technique should be used in any patient will be made in the cardiac catheterization laboratory (15). Abbas et al. revealed that higher TIMI scores (>4) in patients were associated with a greater extent of CAD (16). This classification not only aids in the management of limited resources but also helps avoiding unnecessarily imposing adverse outcomes of invasive techniques on patients at lower risk. On the other hand, an invasive strategy, namely routine coronary angiography, is recommended for patients at high or moderate risk. The results of our study are compatible with the findings of the study by Mega et al. (17), who concluded that the mean TIMI Risk Score of 3 reflects a higher risk. They furthermore showed that despite similar TIMI Risk Scores, in-hospital outcomes in patients with ACS have improved over time (1998 through 2000), and correlated this change with the greater use of newer antiplatelet and antithrombotic therapies, such as glycoprotein and low molecular weight heparin, and the increased availability of intracoronary stenting. Also, Roffi concluded that patients with risk scores of more than 4 were more likely to have critical stenosis (81% vs 58%, *p*<0.001) and multi-vessel disease (80% vs 43%, *p*<0.001) (7). 

It seems that, TIMI score enables us to predict the presence of significant coronary artery lesions, as well as the severity of stenosis and prognosis of patients undergoing thrombolytic therapy. Furthermore, it can help us reap the benefits of early intervention therapy in patients. 

**Table1:** Baseline characteristics of the studied patients

**Variables**		**TIMI score**			**P**
	**Total n=297**	**0-2 n=105**	**3-4 n=120**	**5-7 n=72**	
**Sex**					
Male	173 (58.2)	67 (63.8)	65 (54.2)	41 (56.9)	0.33
Female	124 (41.8)	38 (36.2)	55 (45.8)	31 (43.1)	
**Diagnosis**					
Unstable angina	193 (65.0)	84 (80.0)	77 (64.2)	32 (44.4)	< 0.001
NSTEMI	104 (35.0)	21 (20.0)	43 (35.8)	40 (55.6)	
**CAD risk factor**					
Diabetes mellitus	112 (37.7)	18 (17.1)	56 (46.7)	38 (52.8)	< 0.001
Smoking	134 (45.1)	49 (46.7)	52 (43.3)	33 (45.8)	0.87
Hypertension	183 (61.6)	48 (45.7)	75 (62.5)	60 (83.3)	< 0.001
Dyslipidemia	159 (53.5)	43 (41.0)	64 (53.3)	52 (72.2)	< 0.001
Family history of CAD	75 (25.3)	19 (18.1)	32 (26.7)	24 (33.3)	0.06
**Ectatic vessels**					
Number	26 (8.8)	12 (11.4)	12 (10.0)	2 (2.8)	0.11

Data are presented as number (%). CAD: coronary artery disease; NSTEMI: non-ST segment elevation Myocardial infarction.

**Table 2:** Frequency of TIMI risk score variables in the study population

Age of 65 years or older	116 (39.1)
ST-segment deviations ≥0.05 mV on initial ECG on admission	136 (45.8)
Presence of ≥ 2 episodes of angina 24 hours before the presentation	225 (75.8)
Aspirin use in the past seven days	185 (62.3)
Previous history of coronary stenosis of 50% or more	46 (15.5)
Elevated serum cardiac markers of necrosis	103 (34.7)
Presence of at least three risk factors for coronary artery disease	136 (45.8)

Data are presented as number (%). ECG: electrocardiogram.

**Table 3:** Correlation between TIMI score and number of coronary arteries involved

Involvement*	**Total**	**TIMI score**			**P**
		**0-2 **n=105	**3-4 **n=120	**5-7 **n=72	
Normal/nonsignificant	62 (20.9)	53 (50.5)	9 (7.5)	0 (0.0)	< 0.001
One vessel	68 (22.9)	32 (30.5)	34 (28.3)	2 (2.8)	< 0.001
Two vessels	49 (16.5)	8 (7.6)	28 (23.3)	13 (18.1)	< 0.001
Three vessels	118 (39.7)	12 (11.4)	49 (40.8)	57 (79.2)	< 0.001

Data are presented as number (%). *: number of coronary artery vessels involved in angiography.

## 5. Limitation

There are several limitations to our study. The results of the study are significant; however, larger cohorts are required to assess the accuracy of the TIMI score in predicting the severity of obstructed vessels. The study does not include short or long-term follow-ups, which can develop stronger data about the prognostic aspect of TIMI score. Finally, our results are derived from an observational study on the limited population of patients referring for angiography, and may not apply to the entire population of patients. In addition, our analyses were not focused on mortality.

## 6. Conclusion:

Based on the findings of present study, there is a direct and significant correlation between TIMI score and the number of coronary vessels involved in patients presenting to emergency department following UA or NSTEMI.

## 7. Declaration:

### 7.1. Acknowledgment

None.

### 7.2. Authors' contribution

Conceptualization: Mohammad Hasan Namazi. 

Data curation: Seyede Salimeh Mazloomi, Mohammad Kalate Aghamohammadi.

Formal analysis: Seyede Salimeh Mazloomi, Mohammad Kalate Aghamohammadi.

Methodology: Mohammad Hasan Namazi, Seyede Salimeh Mazloomi.

Project administration & Supervision & Validation: Mohammad Hasan Namazi.

Writing – original draft, review & editing: All authors.

### 7.3. Funding

No funding was received for this study.

### 7.4. Conflict of interest

No potential conflict of interest was reported by the authors.

## References

[1] Anderson JL, Morrow DA (2017). Acute myocardial infarction. New England Journal of Medicine..

[2] Yeh RW, Sidney S, Chandra M, Sorel M, Selby JV, Go AS (2010). Population trends in the incidence and outcomes of acute myocardial infarction. New England Journal of Medicine..

[3] Deaton C, Froelicher ES, Wu LH, Ho C, Shishani K, Jaarsma T (2011). The global burden of cardiovascular disease. European Journal of Cardiovascular Nursing..

[4] White HD, Chew DP (2008). Acute myocardial infarction. The Lancet..

[5] Widimsky P, Wijns W, Fajadet J, De Belder M, Knot J, Aaberge L (2010). Reperfusion therapy for ST elevation acute myocardial infarction in Europe: description of the current situation in 30 countries. European heart journal..

[6] Damman P, Woudstra P, Kuijt WJ, Kikkert WJ, HOEF TP, Grundeken MJ (2013). Short- and long-term prognostic value of the TIMI risk score after primary percutaneous coronary intervention for ST-segment elevation myocardial infarction. Journal of interventional cardiology..

[7] Roffi M, Patrono C, Collet JP, Mueller C, Valgimigli M, Andreotti F (2016). 2015 ESC Guidelines for the management of acute coronary syndromes in patients presenting without persistent ST-segment elevation: Task Force for the Management of Acute Coronary Syndromes in Patients Presenting without Persistent ST-Segment Elevation of the European Society of Cardiology (ESC). european heart journal..

[8] Rao SS, Agasthi P Treasure Island (FL). https://www.ncbi.nlm.nih.gov/books/NBK556069/?report=classic.

[9] Antman EM, Cohen M, Bernink PJ, McCabe CH, Horacek T, Papuchis G (2000). The TIMI risk score for unstable angina/non–ST elevation MI: a method for prognostication and therapeutic decision making. JAMA..

[10] Morrow DA, Antman EM, Snapinn SM, McCabe CH, Theroux P, Braunwald E (2002). An integrated clinical approach to predicting the benefit of tirofiban in non-ST elevation acute coronary syndromes. Application of the TIMI Risk Score for UA/NSTEMI in PRISM-PLUS. Eur Heart J.

[11] Puymirat E, Bonaca M, Fumery M, Tea V, Aissaoui N, Lemesles G (2019). Atherothrombotic risk stratification after acute myocardial infarction: the Thrombolysis in Myocardial Infarction Risk Score for Secondary Prevention in the light of the French registry of acute ST elevation or non‐ST elevation myocardial infarction registries. Clinical cardiology..

[12] Cantor WJ, Goodman SG, Cannon CP, Murphy SA, Charlesworth A, Braunwauld E (2005). Early cardiac catheterization is associated with lower mortality only among high-risk patients with ST-and non–ST-elevation acute coronary syndromes: Observations from the OPUS-TIMI 16 trial. American heart journal..

[13] SociedadeBrasileiraDeCardiologia (2007). Guidelines for unstable angina and Non-ST-Segment elevation myocardial infarction of the brazilian society of cardiology (II Edition, 2007). Arquivos brasileiros de cardiologia..

[14] Terkelsen CJ, Lassen JF, Nørgaard BL, Gerdes JC, Jensen T, Gøtzsche LB (2005). Mortality rates in patients with ST-elevation vs non-ST-elevation acute myocardial infarction: observations from an unselected cohort. European heart journal..

[15] Garcia S, Canoniero M, Peter A, De Marchena E, Ferreira A (2004). Correlation of TIMI risk score with angiographic severityand extent of coronary artery disease in patients with non–ST-elevation acute coronary syndromes. The American journal of cardiology..

[16] Abbas S, Siddiqui AH, Cheema A, Abbas A, Jaffri SK, Khan S (2020). ASSOCIATION OF THROMBOLYSIS IN MYOCARDIAL INFARCTION (TIMI) RISK SCORE WITH EXTENT OF CORONARY ARTERY DISEASE IN PATIENTS WITH UNSTABLE ANGINA AND NSTEMI. Pakistan Armed Forces Medical Journal..

[17] Almeda FQ, Hendel RC, Nathan S, Meyer PM, Calvin JE, Klein LW (2003). Improved in-hospital outcomes in acute coronary syndromes (unstable angina/non-ST segment elevation myocardial infarction) despite similar TIMI risk scores. The Journal of invasive cardiology..

